# New Approaches in the Exploration of Parkinson’s Disease

**DOI:** 10.3390/brainsci15121314

**Published:** 2025-12-09

**Authors:** Tommaso Ercoli, Francesco Loy, Carla Masala, Paolo Solla

**Affiliations:** 1 Department of Neurology, University of Sassari, Viale S. Pietro 10, 07100 Sassari, Italy; psolla@uniss.it; 2 Department of Biomedical Sciences, University of Cagliari, SP8 Cittadella Universitaria, 09042 Monserrato, Italy; floy@unica.it (F.L.); cmasala@unica.it (C.M.)

Parkinson’s disease (PD) is the second most common neurodegenerative disorder, and it is considered one of the major challenges in contemporary neuroscience [1]. Traditionally defined by its cardinal motor manifestations (e.g., tremor, bradykinesia, rigidity, and postural instability), PD is now widely recognized as a complex, multisystem condition with a broad spectrum of non-motor symptoms [2]. PD is also characterized by a long prodromal phase, during which substantial pathological changes precede the onset of the motor presentation [2]. Despite significant progress in understanding its underlying mechanisms, crucial aspects of PD pathogenesis, early detection, and disease-modifying treatment remain unresolved [3]. The exploration of innovative diagnostic methods, refined biomarkers, and novel therapeutic strategies is essential to advance our ability to characterize, monitor, and eventually modify the course of the disease [4,5,6].

Within this context, in this Special Issue entitled “New Approaches in the Exploration of Parkinson’s Disease”, we aimed to expand the current knowledge about PD and other parkinsonian syndromes, focusing on both motor and non-motor symptoms that can negatively impact health-related quality of life [7,8]. To this end, this Special Issue brings together a compelling collection of studies that advance our understanding of PD and related neurological conditions from diverse perspectives. For this purpose, we provided an overview of the nine original high-quality scientific papers included in the aforementioned Special Issue collection.

A prominent theme emerging from these studies is the growing recognition of the significance of molecular and metabolic pathways in PD pathogenesis. The article by Lu and colleagues (Contribution 1) explores the role of Cathepsin B and suggests a potentially protective mechanism mediated through N-acetylaspartate, offering fresh perspectives on lysosomal dysfunction in PD. When viewed alongside these molecular findings, the lifetime evolution model of PD provides a broader framework that incorporates the cumulative environmental, genetic, and biological factors shaping the disease.

A detailed examination of vocal features as potential biomarkers is provided in the review by Wright and Aharonson (Contribution 2), who demonstrate that changes in speech production, articulatory precision, and prosodic patterns can reliably track disease progression. Such developments represent an important step forward, helping to address the limitations of traditional clinical scales and the growing need for reliable, real-time monitoring in a chronic and progressive condition like PD.

The review by Birreci and colleagues (Contribution 3) in this Special Issue provides an overview of the main non-invasive brain stimulation techniques and illustrates how these approaches can modulate neurophysiological mechanisms relevant to Parkinsonism. By summarizing current evidence, the authors highlight the potential of non-invasive brain stimulation to advance biomarker discovery and to support both diagnostic and therapeutic applications. Despite encouraging findings, they also emphasize the need for further methodological refinement and research to fully clarify the role of these techniques in PD and related disorders.

The study by Matsuda and colleagues (Contribution 4) evaluates a short, two-week rehabilitation program in patients with progressive supranuclear palsy (PSP). Their findings show measurable improvements in balance and specific gait parameters, in both the Richardson’s syndrome and progressive gait freezing subtypes, supporting the value of targeted and short-term rehabilitation even in this challenging condition.

A practical approach to describe cognitive heterogeneity in early PD is provided by Álvarez-Avellón and colleagues (Contribution 5) using integrating detailed neuropsychological profiles with visual MRI ratings. The authors identify several distinct anatomo-cognitive subtypes linked to specific patterns of regional atrophy, providing a useful framework that may enhance early patient stratification and support more personalized management strategies.

The study by Siva and colleagues (Contribution 6) shows that PD patients with severe hyposmia exhibit marked alterations in brain network topology compared to those with preserved olfaction and healthy controls. Reduced segregation, efficiency, and small-worldness, together with changes in key networks, suggest that these topological measures may help distinguish PD subgroups.

Moreover, Marano and colleagues (Contribution 7) examine how driving performance changes across motor states in PD. Using a driving simulator, the authors show that patients perform worse than healthy controls, with clear deterioration during wearing-off. They also report that add-on therapies, particularly opicapone, may help preserve specific driving skills. In our Special Issue, the role of levodopa in distinguishing PD from other atypical parkinsonian syndromes, such as multiple system atrophy (MSA) and PSP, is also evaluated. Within this context, Ye and colleagues (Contribution 8) analyzed the individual motor responses to the acute levodopa challenge in PSP and MSA. Despite the overall limited responsiveness, the authors show that specific symptoms (e.g., bradykinesia, rigidity, and tremor) may still improve in some patients, underscoring the heterogeneity of atypical parkinsonism and the need for more personalized therapeutic strategies.

The contribution by Janssen Daalen and colleagues (Contribution 9) reflects on how a lifetime view of Parkinson’s disease—one that considers the long pre-diagnostic phase and early-life risk and protective factors—could help reshape disease-modifying trial design. The authors discuss how this perspective may influence which patients are selected, which mechanisms are targeted, and which biomarkers are most informative. They also underline the importance of closer patient–clinician collaboration when designing future studies.

In conclusion, the contributions collected in this Special Issue highlight how progress in molecular biology, neurophysiology, clinical assessment, and therapeutics continues to broaden our understanding of PD and related parkinsonian syndromes. One of the main goals of this Special Issue was to provide new insights to guide future research initiatives. As Guest Editors, we would like to thank all the authors and reviewers that have participated in this project and the editorial team of Brain Sciences for their constant support.
